# Factors that motivate and hinder blood donation in Greece

**DOI:** 10.1111/j.1365-3148.2007.00797.x

**Published:** 2007-12

**Authors:** O Marantidou, L Loukopoulou, E Zervou, G Martinis, A Egglezou, P Fountouli, P Dimoxenous, M Parara, M Gavalaki, A Maniatis

**Affiliations:** * Blood Bank, Asklipeion HospitalAthens, Greece; † NASA Ames Research Center, San Jose State University FoundationSan Jose, CA, USA; ‡ Blood Bank, University Hospital of IoanninaIoannina, Greece; § Blood Bank, University Hospital of AlexandroupolisAlexandroupolis, Greece; ¶ Blood Bank, Papanikolaou HospitalThessaloniki, Greece; ** Blood Bank, University Hospital of HrakleioCrete, Greece; †† Blood Bank, Mamatseio HospitalKozani, Greece; ‡‡ Blood Bank, Euaggelismos HospitalAthens, Greece; §§ Blood Bank, Laikon HospitalAthens, Greece; ¶¶ Haematology Clinic, Henry Dunan HospitalAthens, Greece

**Keywords:** blood, incentives, perception, replacement, risk, volunteer

## Abstract

Donations in Greece are insufficient to cover the high transfusion needs arising from large numbers of thalassaemia and sickle cell anaemia patients and the implementation of new surgical techniques. Efforts to achieve self-sufficiency, and to render blood supplies safer and manageable must focus on recruiting and retaining more volunteer donors and on converting the large pool of replacement donors. The aim of the study was to gain insight into public perception regarding the risks of donation and transfusion and to identify the factors that would motivate more people in Greece to regularly donate blood. Questionnaires were distributed to 1600 donors at the blood bank and visitors to hospitals at 11 locations across the country. Data on demographics, donation behaviour, incentives, risk perception and attitudes towards donation and transfusion were analysed separately for volunteer and replacement donors and non-donors. The results showed that women and young people donate the least in Greece. Also, many donors do not donate because they are not reminded to. A small percentage of donors confessed to having concealed part of the truth to background questions. Overall, incentives to donate were considered important and included future availability of blood for self or family, paid leave from work and free blood tests. Recruitment and retention efforts should include better communication with current donors, and raising awareness among eligible donors. Staff should be educated in soliciting information from potential donors, and incentives should be better aligned to avoid conflict with ethical values and ensure honesty in the prescreening process.

Efforts continue worldwide to establish and maintain sufficient numbers of regular, volunteer blood donors to ensure an adequate and safe blood supply. The constant concern with being able to meet the demands for blood is because of the fact that only a small percentage of the eligible population actually chooses to donate blood on a regular basis and that a significant percentage of eligible donors are deferred temporarily or permanently because of strict deferral criteria continuously being added in the name of blood safety (Custer *et al*., 2004; Riley *et al*., 2007). At the same time, the demand for blood and blood products in most countries continues to increase because of the rise in human life expectancy and the implementation of new and aggressive surgical and therapeutic methods requiring large quantities of blood and blood products (Provan, 1999; Gillespie & Hillyer, 2002; Currie *et al*., 2004; Greinacher *et al*., 2007; Mathew *et al*., 2007).

The fragile balance between blood supply and demand forces blood banks to constantly search for more efficient ways to recruit blood donors (Ferguson, 1996). The method each country adopts to attract blood donors and to cover its needs in blood supplies varies as a function of its socio-economic structure (Rouger & Hossenlopp, 2005). In spite of all research on the subject, however, the incentives that would motivate most people to become blood donors have yet to be determined (Boulware *et al*., 2002).

The blood donation system in Greece is decentralized and consists of 95 hospital blood banks under the supervision of the Ministry of Health. Each bank is an integrated part of a public hospital and has the responsibility for recruiting blood donors, for collecting and testing blood and for processing it into its products to supply the hospital clinics. It is important to note that a new law designed to organize a centralized system in accordance with the EC directive 2002/98/EC, should be implemented by 2007.

According to data collected by the Ministry of Health, in the year 2005, there were 610 056 blood donations in Greece, of which 322 370 (52·84%) were provided by replacement donors (RDs) who donated blood to cover the transfusion needs of relatives and friends and 270 534 (44·35%) by volunteer donors (VDs) who donated on their own initiative. A small number, 17 152 (2·81%), of donors belonged to the armed forces. This last group is highly motivated to donate voluntarily as they are compensated with days off from duty. Despite these figures, however, and the slight increase in blood donations in 2005 compared with previous years, 24 000 red blood cell units were imported from Switzerland to cover the national needs for blood.

There are a number of reasons why Greece, like many countries, would like to increase the number of regular VDs. As stated above, the number of donations is insufficient to cover the demand leading to the importation of blood from abroad almost every year. Also, VDs are generally associated with safer blood supplies in terms of transfusion-transmitted diseases (Maniatis *et al*., 1994; Liu *et al*., 1998). In fact, the World Health Organization and the Council of Europe recommend that blood and blood components should only be collected from voluntary, non-remunerated repeat donors (Dhingra, 2002; Council of Europe, 2003). In turn, blood donation systems that rely on volunteer blood donors who donate on a regular basis can better manage blood supplies and schedule transfusions. Finally, it is inappropriate, from an ethical point of view, that relatives of a patient in need of blood should, under emotional pressure, be obliged to search for people willing to donate blood for their relative's transfusion needs.

Thus, the effort of the blood donation system in our country has two goals: (1) the overall increase of blood units collected to ensure self-sufficiency in blood supply and (2) the conversion of RDs into regular, VDs to increase the safety and facilitate the management of the available blood supply.

We report on a questionnaire study designed to identify the factors that motivate or discourage blood donation among the Greek population and to evaluate perceptions about the risks of donation and transfusion. Data are compared between VDs, RDs and non-donors (NDs).

## Materials and methods

The study was conducted by the Hellenic Blood Transfusion Society between September 2005 and March 2006 with the participation of 11 hospital blood banks representing all the geographical regions of the country. A questionnaire was distributed twice each week (on specific days) to every person 18 years of age or older who had just finished donating blood at the blood bank, and to randomly selected visitors of the hospital's patients. Questionnaires were completed anonymously and deposited in a special box by the respondents.

The questionnaire consisted of multiple choice and rating questions and required approximately 15 min to complete. Each questionnaire contained an introduction explaining the purpose of the survey and provided the definitions of VD, RD, transfusion and transfusion alternatives. The questionnaire contained some questions directed to both donors and NDs, and other questions directed to each group separately.

In general, the questions concerned:

1. demographic characteristics (age, sex, education, etc.);
2. donor and ND behaviour (total number of donations, reasons for donating, reasons for not donating, etc.);
3. predonation screening questionnaire;
4. donor satisfaction with services at donation locations;
5. factors that motivate or discourage blood donation (incentives);
6. level of knowledge about blood donation and transfusion;
7. risk perception (perceived risks, fears);
8. attitudes about blood transfusion.

### Statistical analysis

All data are presented in tables of the distribution of responses to specific questions by demographic or respondent group classification. The differences among and within the three respondent groups were assessed using the χ^2^ test or Fisher's exact test (when the expected frequency in any cell was below 5) (Everitt, 1992). In general, there was full concordance between the χ^2^ test and Fisher's exact test *P* values, thus only the Fisher's exact test *P* values are reported in the tables.

## Results

### Demographic characteristics

The questionnaire was completed by 1600 respondents, of whom 898 (56·1%) were men and 702 (43·9%) were women. Questionnaires were distributed almost evenly among 880 (55·0%) individuals at blood banks who had just finished donating blood (67·7 men and 32·3% women) and 720 (45·0%) hospital visitors (42·0 men and 58·0% women).

In total, 1136 (71·0%) respondents were donors, of which 579 (51·0%) were VDs and 557 (49·0%) were RDs and 464 (29·0%) were NDs (Table 1). Gender distribution within the different respondent groups was different for VDs (66·7 men and 33·3% women), RDs (70·5 men and 29·5% women) and NDs (25·8 men and 74·2% women). These distributions were statistically significant between RDs and NDs (*P* < 0·0001), but not VDs. Overall, more men than women surveyed were donors (VDs or RDs). Of the hospital visitors, 256 (35·5%) were donors and 464 (64·5%) were NDs, and again more men than women were donors (71·5 and 28·5%, respectively).

**Table 1:** Distribution of respondent groups by gender

	% (*n*)	% (*n*)	% (*n*)	% (*n*)
	VDs	RDs	NDs	Total
All respondents				
Gender				
Male	66·7 (386)	70·5 (392)	25·8 (120)	56·1 (899)
Female	33·3 (193)	29·5 (165)	74·2 (344)	43·9 (701)
Total	100·0 (579)	100·0 (557)	100·0 (464)	100·0 (1600)
*P* value	0·2473	<0·0001	<0·0001	<0·0001
		Donors	NDs	Total
Hospital visitors only				
Gender				
Men		71·5 (183)	25·8 (120)	42·0 (303)
Women		28·5 (73)	74·2 (344)	58·0 (417)
Total		35·5 (256)	64·5 (464)	100·0 (720)

Large differences were observed in the distribution of respondents according to age (Table 2). Almost half (49·3%) of NDs were 18–30 years old (*P* < 0·0001). This proportion was only 33·5% among VDs (*P* = 0·1768) and 25·2% among RDs (*P* < 0·001).

**Table 2:** Distribution of respondent groups by age

	% (*n*)			
	
Age group	VDs	RDs	NDs	Total
18–30	33·5 (194)	25·2 (140)	49·3 (229)	35·2 (563)
31–40	34·6 (200)	39·0 (217)	21·1 (98)	32·2 (515)
41–50	22·7 (132)	25·0 (139)	19·8 (92)	22·7 (363)
51–60	8·3 (48)	9·9 (56)	6·9 (32)	8·5 (136)
> 60	0·9 (5)	0·9 (5)	2·7 (13)	1·4 (23)
Total	100·0 (579)	100·0 (557)	100·0 (464)	100·0 (1600)
*P* value	0·1768	<0·0001	<0·0001	<0·0001

### Total number of donations

Overall, VDs reported giving blood more often than RDs (*P* < 0·0001). Indeed, 41·1 % of VDs gave blood more than 10 times and 29% of them—one to three times, whereas the corresponding percentages for RDs were 23·7 and 42·2% (Table 3).

**Table 3:** Total number of donations among donors (VDs and RDs)

	% (*n*)			
		
Total number of donations	VDs	RDs	Total	*P* value
1–3 times	29·0 (168)	42·2 (235)	35·4 (403)	<0·0001
4–10 times	29·9 (173)	34·1 (190)	31·9 (363)	<0·0001
>10 times	41·1 (238)	23·7 (132)	32·7 (370)	<0·0001

### Reasons for not having donated blood for more than a year or for never having donated blood

Almost half (46·3%) of the donors, most of them RDs (55·4% RDs compared with 37·6% VDs, *P* < 0·0001) who reported having donated blood at least once in the past, stated that they had not donated in the past 12 months (Table 4). When asked for the reasons, 39·1% of them (54·6% RDs and 16·5% VDs) reported that they had not been asked to (*P* < 0·0001) and 21·3% of them (15·2% RDs and 30·3% VDs) reported that they had health problems (*P* < 0·0001) (the questionnaire did not specify what type of problems). Secondary reasons were the lack of time (11·6% RDs and 18·1% VDs) and having been rejected as donors (9·8% RDs and 11·2% VDs).

**Table 4:** Reasons for not having donated blood ever on in more than a year

	% (*n*)			
		
	VDs	RDs	Total	*P* value
I have not donated in more than a year	37·6 (218)	55·4 (309)	46·3 (527)	<0·0001
Reasons				
Health problem	30·3 (175)	15·2 (87)	21·3 (262)	<0·0001
I was rejected as a donor	11·2 (65)	9·8 (55)	10·3 (120)	0·6440
I was not asked to	16·5 (96)	54·6 (304)	39·1 (400)	<0·0001
I do not have time	18·1 (105)	11·6 (65)	14·2 (170)	0·0580
Other reasons	6·5 (38)	4·7 (26)	5·4 (64)	0·5301
I have never donated				
Reasons	NDs			
Health problem	37·7 (175)			
I was rejected as a donor	18·2 (84)			
No one in immediate environment needed it	20·2 (94)			
I was not asked to	21·6 (100)			
I do not have time	2·0 (9)			

When asked why they had never donated blood, NDs reported health problems (37·7%), not having been asked to (21·6%) and that no one in their immediate environment had needed it (20·2%) as the main reasons.

### Initial donation

Most VDs (85·5%) gave blood for the first time voluntarily. Only 7·5% of them initially donated blood for a friend or relative in need, i.e. were RDs before choosing to become VDs. In contrast, the overwhelming majority (77·3%) of RDs began giving blood as RDs and only 20·2 % started out as VDs (*P* < 0·0001) (Table 5). It is worth noting that 7·0% of VDs and 2·5% of RDs admitted to donating blood for the first time in order to earn paid leave from work.

**Table 5:** Reasons for initially donating blood

	% (*n*)			
		
	VDs	RDs	Total	*P* value
For relative/friend	7·5 (43)	77·3 (431)	44·6 (474)	<0·0001
Voluntarily	85·5 (495)	20·2 (113)	50·8 (608)	<0·0001
To earn paid leave	7·0 (41)	2·5 (14)	4·6 (55)	0·0015

### Predonation screening

Although the majority of respondents from all three groups stated that they believe there are no indiscreet questions in the predonation screening questionnaire (92·0%) and that all questions are important for the safety of the patient and themselves (97·0%), 5·3% of all donors admit to having concealed part of the truth when responding to background questions (Table 6). Of this percentage, 3·4% were VDs and 7·1% were RDs and this difference was statistically significant (*P* < 0·02). As a reason for having concealed the truth, 69·2% of VDs and 20·0% of RDs confessed to not thinking the particular question was of importance (*P* < 0·0001), 30·8% of VDs and 10% of RDs to earn paid leave from work and 7·7% of VDs and 3·3% of RDs to benefit from the free blood tests (such tests are for cholesterol, triglycerides, etc. and do not include testing for infectious diseases). Of interest is the fact that 80% of the RDs who reported having concealed the truth when describing their medical history confessed to having done so to be accepted in order to ensure their relatives’ needs were covered.

**Table 6:** I have hidden the truth in the predonation screening questionnaire

	% (*n*)			
		
	VDs	RDs	Total	*P* value
I have hidden the truth	3·4 (20)	7·1 (40)	5·3 (60)	0·0155
Reasons				
To earn paid leave	30·8 (6)	10·0 (4)	16·6 (10)	0·1723
To ensure relative's needs are covered	0·0 (0)	80·0 (32)	53·3 (32)	<0·0001
To benefit from free blood tests	7·7 (2)	3·3 (1)	5·0 (3)	0·5183
I did not think the question was important	69·2 (13)	20·0 (8)	34·9 (21)	<0·0001

### Donor satisfaction with services at donation locations

Most of the donors (97·0%) reported being satisfied with the services and attitude of the staff. They were, however, displeased by the waiting time (48·7%) and the unpleasantness of the physical environment (32·3%).

### Factors that motivate or discourage blood donation

The majority of all three respondent groups (>99·0%) stated that they believe that blood donation is an important contribution to their fellow human beings. In answering a question about whether there should be incentives in place for volunteer donation, a large majority [76·5, 77·4 and 73·8% (*P* = 0·4865) of VDs, RDs and NDs, respectively] agreed that such incentives should exist and stated that their preferred incentives are (a) future blood availability for themselves and their families when needed (85·1% of VDs, 86·5% of RDs and 78·6% of NDs; *P* = 0·0191), (b) paid leave from work (40% of VDs, 37·2% of RDs and 43·5% of NDs; *P* = 0·2655) and (c) free blood testing for cholesterol, triglycerides, etc. (39·9% of VDs, 35·8% of RDs and 38·3% of NDs; *P* = 0·5113).

### Level of knowledge about blood donation and transfusion

More than 95·0% of all three groups of respondents agreed that they are aware that there is a shortage of blood in Greece and responded correctly to most questions regarding blood donation and transfusion. Overall there appeared to be good awareness of basic blood donation facts (e.g. age at which one can donate, acceptable duration of storage of blood and tests performed on donated blood).

### Risk perception

#### Blood donation

Respondents rated the degree (none, small, medium and high) to which they agreed that they are personally subject to each of many commonly cited risks when donating blood. None of the respondents rated any of the items as ‘high’ (i.e. that they felt they ran a high risk). Most risks were rated as ‘small”’. Responses did not vary significantly among NDs and donors concerning the fear of becoming anaemic, although NDs rated the fear of fainting, feeling weak or dizziness higher than donors did (38·5 vs. 23·0%, respectively) (*P* < 0·0001). The three groups did not differ in their ratings of risk of becoming infected with transmissible diseases such as human immunodeficiency virus (HIV)/hepatitis, or their fear of the results of blood tests. Risks were rated as ‘none’ and ‘slight’.

#### Blood transfusion

Eighty per cent of all respondents believe that there is only a ‘small’ risk of being transfused with blood from the wrong blood group. On the contrary, whereas more than half of all respondents believe that the risk of becoming infected with HIV or hepatitis B virus (HBV) is ‘small’ (69·0 and 57·0% of respondents, respectively), 14·0% of them believe that the same risk for either of the two viruses is ‘medium’. About 9·0% of all respondents believe that the risk of becoming infected is ‘large’. There were no statistically significant differences among the three respondent groups.

### Attitudes about blood transfusion

The majority (96·0%) of all three groups of respondents believe that patients must be informed before being transfused. A significant number (59·8% of VDs, 56·7% of RDs and 49·4% of NDs) (*P* < 0·001) stated that they believe patients should be informed about the benefits of a transfusion. A smaller number (40·5, 43·1, and 43·1 of VDs, RDs and NDs, respectively) (*P* = 0·6994) stated that they believe patients should be informed about alternatives to transfusion, when these exist. Almost everyone (85·0%) believes that patients should have the right to refuse a transfusion, if alternatives exist.

## Discussion

Despite having a blood donor index of six blood donors per 100 citizens and ranking second among European Union member states in the number of people who have donated blood at least once in the past (Eurobarometer 2003), and although being first in the proportion of NDs who have at least considered giving blood (Eurobarometer 2005), Greece with a population of approximately 11 000 000, often finds the number of annual blood donations insufficient to cover the high national transfusion needs and some blood is imported. This is largely because of the fact that about 20% (120 000) of the annually issued blood units are required for the transfusion of approximately 3000 patients suffering from thalassaemia and sickle cell anaemia. In addition, the need for blood during various surgical procedures is larger than that in other Central and Northern European countries as revealed by the safe and good use of blood (SANGUIS) study (Sirchia *et al*., 1994) and efforts to minimize such blood usage have not been effective.

Given the country's yearly inability to achieve self-sufficiency, and in the effort to increase the number of donors, it is important to consider the source of blood donations. Less than half of these (44·35%) came from VDs and 52·84% came from patients’ relatives. The remaining came from the armed forces (motivated by days off from duty and hence not likely to become regular, VDs) or were imported from abroad.

The large proportion of RDs means everyday difficulties in managing blood reserves that, in turn, translates into psychological pressure on the relatives of patients to locate donors so that their relative/friend can be transfused. In addition, RDs may increase the risk of transfusion-transmitted diseases in the blood supply. According to the Hellenic Coordinating Haemovigilance Centre data from 1999, 75·0, 87·8, and 87·0% of all donors that tested positive for HBV, hepatitis C virus and syphilis, respectively, were RDs (SKAE, 2005).

It is clear that a much larger number of regular, VDs is required to ensure and manage an adequate and safe blood supply in the country. The results of the present study reveal interesting facts regarding public behaviour and perception towards blood donation and blood transfusion. If used effectively, such results can ultimately help in the effort to attract and retain more VDs in general, and to convert the currently large pool of RDs into VDs. This will enable correct scheduling and adequate supplies of safer blood and blood products. Selected findings are discussed here as they relate specifically to the design of national recruitment and retention strategies, as well as to approaches for converting RDs into VDs.

The data of this study show that women and young people are the two groups that donate the least. Women generally tend to donate blood less often than men because of medical reasons. Many women do not volunteer to donate because they think they are ineligible or have actually been rejected once because of low body weight and because they are prone to anaemia, especially during their childbearing age due to their increased need for iron. At the New York blood centre, for example, 92·7% of the donors deferred for low haemoglobin level were women (Danvey, 2004). We can therefore increase the number of women donors via administration of iron supplements combined with proper and personalized monitoring and support, as indicated by several studies (Gordeuk *et al*., 1990; Hartsfield, 1998; Alvarez-Ossorio *et al*., 2000; Newman, 2004). Such a measure must be accompanied by the appropriate information, explaining to women that they are not being rejected, only temporarily deferred and encouraging them to return when their haemoglobin has returned to normal values (Bianco *et al*., 2002).

As far as young people are concerned, it looks like we have failed to create appropriate recruitment programmes to date, and we will have to further study and understand the motives that attract young people. After all, the failure to attract VDs of a young age is a problem in other countries too (Lemmens *et al*., 2005; Misje *et al*., 2005).

Despite the fact that almost all participants from the three groups believe that blood donation is an important contribution to their fellow citizen, that there is a shortage of blood supply in Greece, that it is difficult to find blood when they need it and despite the fact that they are satisfied by the services at blood donation centres, a large percentage of VDs and an even larger proportion of RDs have not given blood in more than a year.

Although the most commonly cited reason for volunteers to not have donated are health problems (this may include rejection due to health problems), other important reasons are time constraints and lack of reminders. RDs have different motivations and most of them reported waiting to donate when someone in their environment is in need. The latter reason is also cited by NDs. Our efforts should therefore definitely ensure that reminding mechanisms are in place and should pay particular attention to reaching eligible blood donors who are willing to donate but simply need to be reminded. Other factors, such as not finding the donation centre hours convenient or the distance of its location do not seem to be significant barriers to blood donation, as described in other studies (Schreiber *et al*., 2006). It is important to know that people in Greece can donate blood at any hospital and donation centres are generally open all day.

Volunteers are more regular donors and donate much larger quantities of blood per person compared with RDs. The largest proportion of VDs, in contrast to the small percentage of RDs, reported having donated more than 10 times. High and regular frequency of donations is generally desirable, as it is connected with an increased level of safety in blood supplies. According to a recent study, the likelihood of donations that test positive for transfusion-transmitted diseases appeared to increase steadily with lapsed interval length (Schreiber *et al*., 2003). One way to increase the frequency of donations is through more effective communication with donors. Our current efforts must be rendered more methodical and accomplished through a wider range of tools (e.g. telephonic or electronic reminders, via television, advertisements and letters).

Donors do not feel that the screening questionnaire contains personal questions regarding their personal life (their sexual preference, etc.). There are, however, both VDs and RDs who admit to having hidden the truth about their background. Similar findings for VDs have been reported elsewhere (Chiavetta *et al*., 2000). The corresponding percentage is higher for RDs than VDs. The reason given by more than half of VDs for having hidden the truth is that they did not consider the question important. In contrast, RDs report hiding the truth to ensure their relative or friend receives the transfusion they need. Such a finding raises concern and highlights the dangers related to replacement blood donations, both in the form of risk to those being transfused as well as risk to the donors. The fact that some people hide the truth about their background shows that more attention must be given to the medical history by educating staff in personalizing the manner in which they solicit information depending on the educational level of the donor.

It must also be noted that a large number of volunteers report having hidden the truth in the screening questionnaire so that they are allowed to donate blood in order to be granted leave from work and to receive free blood testing. This finding must be evaluated in combination with the fact that these are commonly recognized incentives for donation in other countries as well (Sanchez *et al*., 2001).

In answering a question about whether there should be incentives in place for volunteer donations, a large majority of each group agreed that such incentives should exist and stated their preferred incentives to be: (a) future blood availability for themselves if/when needed; (b) paid leave from work (public servants in Greece are granted paid leave from work when they donate blood) and (c) free blood testing (cholesterol, triglycerides, etc.).

Awards and small souvenirs have a smaller impact. The incentives for blood donation that arise from the results of our study are common with those in studies from other countries too (Glynn *et al*., 2003).

Ideally, donating blood is an important act of altruism and should not have to be reinforced by rewards or awards. However, because the volume of volunteer donations is generally insufficient, it has become acceptable practice to rely on rewards. In general, rewards should not be related to material possessions (e.g. monetary compensation), so that there is no conflict with ethical values and individuals are not tempted to be less than honest during the predonation screening in order to obtain a reward.

Free blood testing is a reward that VDs in our country count on. This has also been shown to be useful in recruiting donors in other studies (Murray, 1988).

In addition, our data showed that this type of reward is not strongly associated with hiding the truth in the prescreening questionnaire. Accordingly, it is one incentive that may be further emphasized in recruitment and retention efforts. Earning paid leave from work, however, may be considered less appropriate as a reward and the data show a large number of donors admitting to not being truthful during screening in order to obtain leave. Our efforts should therefore aim to suspend this practice that may lead to increases in at-risk donors. This change must of course be made in stages and must be implemented by education of the public so that it does not negatively affect donor turnout.

Results from the section of the study addressing risk perception indicate that people are concerned, at least to a small degree, about a large range of factors discouraging blood donations (e.g. the fear of needles, of the sight of blood, of feeling pain, of fainting, of developing anaemia, of feeling weakness and fear of infection). These data are similar to that from other studies, for example, fear of the collection process was the dominant factor for avoiding donation among young Canadian college students (Hupfer *et al*., 2005). Overall, NDs in our study appear to have a greater sense of risk than donors. New recruitment efforts should therefore address these risk factors and ensure that the appropriate, realistic perspective is communicated to the public so as to attract new donors.

Our findings from the section of the study that addressed transfusion facts and perceived risks highlighted a problem of inadequate briefing of patients in Greek hospitals about the medical procedures to which they will be subjected and their associated risks. Doctors should be made aware of this issue and the patients’ rights and should be encouraged to communicate with their patients, informing them of the potential need to be transfused, the benefits of transfusion, the possible side effects and risks, and giving them the right to refuse a transfusion when alternatives are available.

## Conclusion

This study revealed the weaknesses of the Greek blood donation system regarding recruitment and retention of donors and the perceptions of people who donate or who could donate blood. We believe that the results provide useful insights that can be used to form plans to encourage current donors to donate more often, to motivate people eligible to donate to support the nation's transfusion needs and to convert the existing body of RDs into VDs.
